# Seed dormancy cycling: A driver of germination timing in a facultative winter annual

**DOI:** 10.1016/j.pld.2025.05.007

**Published:** 2025-05-27

**Authors:** Jingshi Yang, Yan Luo, Jerry M. Baskin, Carol C. Baskin, Andreas Prinzing, Luping Liu, Chaohan Xu, Keliang Zhang

**Affiliations:** a College of Horticulture and Landscape Architecture, Yangzhou University, Yangzhou 225009, China; b Department of Biology, University of Kentucky, Lexington, KY 40506, USA; c Department of Plant and Soil Sciences, University of Kentucky, Lexington, KY 40546, USA; d Research Unit “Ecosystem, Biodiversity, Evolution”, Université de Rennes 1/ Centre National de la Recherche Scientifique, Campus de Beaulieu, Bâtiment 14A, Rennes 35042, France

**Keywords:** Brassicaceae, *Cardamine impatiens*, Conditional dormancy, Dormancy cycling, Light-dependent germination

## Abstract

Timing of seed germination is critical for survival of annual plants in seasonal climates. We tested the hypothesis that seeds of the winter annual species *Cardamine impatiens* (Brassicaceae) “track” their thermal environment and synchronize germination with favorable growth conditions. We predicted that seeds buried in the field from maturity to autumn germinate best at autumn temperatures, while those buried from maturity to spring germinate best in early spring. We monitored seasonal changes in germinability by exhuming field-buried seeds monthly for 30 months and incubating them under laboratory conditions. The effects of temperature on the transition of dormancy status also were investigated. Seeds of *C. impatiens* were dormant at dispersal in May, and during summer dormancy transitioned to conditional dormancy and then to non-dormancy. By early autumn, seeds germinated in a wide range of temperature regimes in light. Nongerminated seeds re-entered conditional dormancy during winter, losing the ability to germinate at high, but not low, temperatures in light. The light requirement for germination was reduced during prolonged seed burial. Overall, our hypothesis is supported. Buried seeds of *C. impatiens* exhibited a seasonally synchronized conditional dormancy/non-dormancy cycle, enabling germination in both autumn and early spring; this information will facilitate management efforts of this weedy species. We conclude that dormancy cycling in *C. impatiens* is an adaptive functional trait that controls the timing of germination, thereby optimizing seedling emergence under favorable conditions while avoiding summer heat.

## Highlights

• Fresh seeds of *C. impatiens* are dormant, and dormancy is broken by high summer temperatures.
• Seeds germinate in the field in both autumn and early spring.
• Seeds failing to germinate in autumn do so only in light at low temperatures in spring.
• Seeds buried in soil exhibit an annual conditional dormancy/non-dormancy cycle.
• Seed dormancy cycling is an adaptive functional trait.

## 1 Introduction

Timing of seed germination is crucial for plant growth/survival (Cao et al., 2012; Lu et al., 2014), plant fitness (Donohue et al., 2005; Leverett et al., 2018), niche construction (Donohue, 2002; 2005), species coexistence (Blackford et al., 2020), community composition (Jiménez-Alfaro et al., 2016; Liu et al., 2020), and ecosystem resilience to climate change (Walter et al., 2020). Based on timing of seed germination, annual plant species of the temperate zone can be categorized into winter annuals and summer annuals (Baskin and Baskin, 2014). Summer annuals germinate in spring or early summer and grow and set seeds at warm temperatures, thus completing their life cycle before autumn frosts (Smith and Weller, 2020). Summer annual plants cannot tolerate low winter temperatures, and the species survives the cold season as seeds that undergo dormancy-break at the low winter temperatures. Seeds are nondormant by spring and can germinate with the onset of warm temperatures (Smith and Weller, 2020). In contrast, seeds of winter annuals germinate in autumn, plants can tolerate low winter temperatures but not summer heat and drought (Cici and Van Acker, 2009; Lu et al., 2016).

Winter annuals that germinate only in autumn are obligate winter annuals, and those that germinate in autumn and in early spring are facultative winter annuals (Cici and Van Acker, 2009; Baskin and Baskin, 2014; Han et al., 2016). Furthermore, winter annuals that germinate in autumn overwinter as rosettes or semi-rosettes, and they may (or may not) require vernalization for flowering (Kim et al., 2009; Matar et al., 2021). Plants from spring-germinated seeds behave as short-lived summer annuals (or spring ephemerals) (*e.g*., Wang et al., 2010; Baskin and Baskin, 2014; Lu et al., 2014). Regardless of whether seeds germinate in autumn or spring, plants complete their life cycle and die before onset of the summer heat (Xu and Chong, 2018; Guo et al., 2025).

The Brassicaceae is one of the most extensively studied families for winter annual species. Freshly matured seeds of obligate winter annuals, such as *Arabidopsis thaliana* (L.) Heynh. (Baskin and Baskin, 1983; Montesinos et al., 2009), *Draba verna* L. (Baskin and Baskin, 1988), and *Lesquerella lescurii* S. Watson (Baskin et al., 1992), as well as facultative winter annuals such as *Capsella bursa-pastoris* (L.) Medik. (Baskin and Baskin, 1989a), *Cardamine flexuosa* With. (Yatsu et al., 2003), *C. hirsuta* L. (Baskin and Baskin, 1988), *Sinapis arvensis* L. (Soltani et al., 2016), and *Thlaspi arvense* L. (Baskin and Baskin, 1989b) are innately dormant (D) and do not germinate at any temperature. In contrast, freshly matured seeds of the obligate winter annual *Alyssum alyssoides* (L.) L*.* (Baskin and Baskin, 1988) and the facultative winter annuals like *Barbarea vulgaris* W.T. Aiton (Baskin and Baskin, 1988) germinated only at low temperatures (10–15 °C) and thus exhibited conditional dormancy (CD). For all these Brassicaceae species, dormancy is broken in summer, and germination occurs when temperatures decrease in autumn especially in light. Seeds that fail to germinate in late autumn enter secondary D or CD in winter. Seeds of obligate winter annuals lose the ability to germinate at high and low temperatures, *i.e.*, become innately D (true dormancy *sensu* Vegis, 1964), while those of facultative winter annuals lose the ability to germinate at high but not low temperatures, *i.e.*, enter CD (Baskin and Baskin, 2014). That is to say, after their innate dormancy is broken (D→CD→ND), seeds of obligate winter annuals exhibit a ND↔CD↔D cycle, whereas facultative winter annuals exhibit a ND↔CD cycle. Thus, seeds of facultative winter annuals, but not obligate winter annuals, can germinate at low spring temperatures.

*Cardamine impatiens* L. (Brassicaceae), also known as narrowleaf bittercress, is commonly found on moist, shaded slopes, streamsides, fields, and roadsides (Pykälä, 1991; Zhou et al., 2001). This species is native from Scotland eastward into northern and central Asia (Williams, 2000; Glenn and Berringer, 2004), but it also has been introduced to North America (Williams, 2000) and Western Europe. It is listed as an invasive species by the Invasive Plant Atlas of New England (Mehrhoff et al., 2003) and exhibits the potential to become an invasive weed in Connecticut (Glenn and Berringer, 2004) and Western Virginia of the United States (Huffman, 2008). *C*. *impatiens* exhibits distinct life forms depending on the region; for instance, it is reported as an annual in “parts of Asia” (Huffman, 2008) and a biennial in the United States, where seeds mature from late May to late August and germinate immediately after being ejected from the plant’s mechanically explosive siliques, *i.e.*, non-dormant (Glenn and Berringer, 2004). In China, *C. impatiens* is a common weed in most provinces (Fig. S1; Zhou et al., 2001). According to our field observations, *C. impatiens* behaves as a facultative winter annual, *i.e.*, seeds germinate in both autumn and early spring. Nevertheless, little is known about the dormancy-breaking and germination requirements or the factors controlling germination timing of this species. Hence, understanding how the environment regulates dormancy and germination of this species is essential both for elucidating its life-history strategies in heterogeneous/disturbed environments and for improving weed management practices and controlling invasions in regions where it is invasive.

We hypothesized that seeds of facultative winter annuals like *Cardamine* *impatiens* can ‘track’ the thermal environment they experience, adjusting (expanding or narrowing) their thermal window for germination accordingly, thereby fine-tuning germination timing to synchronize with autumn and spring. In other words, seeds of *C. impatiens* exhibit a dormancy cycle that serves as an adaptive trait, allowing plants to establish under conditions that likely maximize their fitness. To test this hypothesis, we buried freshly matured seeds of this species in soil and at monthly intervals for 30 months tested germination in light and dark over a range of temperature regimes. We also conducted laboratory studies to determine the temperature requirements for dormancy-break and secondary dormancy induction.

Based on studies of facultative winter annual species of Brassicaceae (*e.g*., Baskin and Baskin, 1988, 1989b; Soltani et al., 2016), we predicted the following. (1) Freshly matured seeds of *Cardamine* *impatiens* are D, with dormancy break in summer and germination in autumn. (2) Buried seeds that fail to germinate in autumn enter CD in winter, losing their germinability at high, but not low, temperatures. (3) The nongerminated seeds undergo repeated cycles between CD↔ND until the cohort of seeds is depleted in the soil seed bank. If our hypothesis is correct, we further predicted that (4) buried seeds exhumed between maturity and autumn germinate best (highest percentage) under autumn-like temperatures, while those exhumed between winter and early spring germinate best under spring temperatures.

## 2 Materials and methods

### 2.1 Seed collection and study site

Freshly matured seeds of *Cardamine* *impatiens* were collected from naturalized populations on the campus of Yangzhou University between 10 and 20 May 2022. Anthropogenic disturbance was not prevented in the populations since the species naturally grows well in disturbed habitats (Pykälä, 1991; Zhou et al., 2001). Seeds were pooled and air-dried at room conditions (18–24 °C, 45–63% relative humidity) for 1 week before germination experiments were initiated. All experiments began within 2 weeks after seed collection, thus avoiding changes in germination behavior (afterripening) during storage (Baskin and Baskin, 2014).

The collection site is geographically located in the eastern and mid-southern parts of the species’ natural distribution range in China (Fig. S1). It has a subtropical monsoon climate characterized by hot summers and cold winters. Mean annual temperature is 16.2 °C, with the highest average temperature in July (28.4 °C) and the lowest average temperature in January (2.8 °C). Annual precipitation is 1066 mm, with 59.1% falling in summer. Extreme temperatures recorded range from −17.7 °C (January 1955) to 41.1 °C (August 2022) [China National Meteorological Data Service, China Meteorological Administration (http://www.cma.gov.cn)]. Soil at the collection sites was yellow-brown soil with loamy texture (Alfisols in US soil taxonomy; Tan et al., 2013), with a pH of 7.50 ± 0.19 (mean ± s.d., measured in March 2020).

The vegetation type on the campus is classified as northern subtropical deciduous and evergreen broad-leaved mixed forest. The species associated with *Cardamine* *impatiens* on the campus included trees: *Celtis sinensis* Pers. and *Metasequoia glyptostroboides* Hu & W.C. Cheng; shrubs: *Osmanthus fragrans* (Thunb.) Lour., *Pittosporum tobira* (Thunb.) W.T. Aiton, and *Chimonanthus praecox* (L.) Link; and herbs: *Cerastium arvense* L., *Draba nemorosa* L., *Erigeron canadensis* L., *Mazus pumilus* (Burm.f.) Steenis, *Medicago lupulina* L., *Poa pratensis* L., *Solanum nigrum* L., *Veronica polita* Fries, and *Viola philippica* Cav.

### 2.2 Overall procedures for germination tests

For all germination experiments, four replicates of 25 seeds each were placed on two sheets of filter paper moistened with 3 mL of distilled water in a 5-cm-diameter Petri dish. The dishes were sealed with Parafilm to slow moisture loss, and additional water was added as needed during incubation to keep the filter paper moist. Germination was tested at 5 °C, 15/5 °C, 20/10 °C, 25/15 °C, 30/20 °C, and 35/25 °C. These thermoperiods correspond to the mean daily maximum and minimum monthly temperatures in the local climate: 15/5 °C, March and November; 20/10 °C, April; 25/15 °C, May and October; 30/20 °C, June and September, and 35/25 °C, July and August. Additionally, a constant 5 °C temperature was used to simulate the low-temperature stratification effect of December, January, and February. Incubations was carried out either under a 12-h photoperiod (∼100 μmol m^−2^ s^−1^, cool white fluorescent light; hereafter ‘light’) or in continuous darkness (with dishes placed in opaque black bags; hereafter ‘darkness’). Germination was defined as the emergence of the radicle (Baskin and Baskin, 2014). At the end of each experiment, nongerminated seeds were examined to determine if they had a grey, soft embryo (non-viable) or a white, firm embryo (viable) (Cao et al., 2014; Zhang et al., 2022). All germination experiments lasted 2 weeks. We note that the daily number of germinated seeds peaked within the first week, and almost no seeds germinated after 10 days. For seeds incubated under light, germination was recorded daily, while for seeds in darkness germination was recorded once at the end of the test. Germination percentages were calculated based on the number of viable seeds.

### 2.3 Effects of light and dry storage period (afterripening) on seed germination

To determine if freshly matured seeds of *Cardamine* *impatiens* were dormant, and if so how afterripening influenced germination, seeds were stored under ambient room conditions for 5 months. Each month from May to October, four replicates of 25 seeds were tested for germination at 5 °C, 15/5 °C, 20/10 °C, 25/15 °C, 30/20 °C, and 35/25 °C, under both light and dark conditions.

### 2.4 Temperature requirement for dormancy break

To determine the temperature requirement for dormancy-break, freshly matured seeds were placed into 30 nylon bags (containing seeds only, without sand). Five bags were placed in each of six metal boxes (20 cm diameter × 10 cm deep) filled with moist sand (11–14% water content), with the bags positioned at a depth of 2 cm. Each of the six boxes was covered with their lids and exposed to one of the following temperature conditions: 5 °C, 15/5 °C, 20/10 °C, 25/15 °C, 30/20 °C, or 35/25 °C for a period of 5 months. After 0, 1, 2, 3, 4, and 5 months, one bag was removed from each temperature, and germination was tested under light conditions at 5 °C, 15/5 °C, 20/10 °C, 25/15 °C, 30/20 °C, and 35/25 °C, *i.e.*, there were six treatment temperatures × six monthly germination tests × six germination temperatures × four replicates × 25 seeds = 21,600 seeds.

### 2.5 Temperature requirement for induction of secondary dormancy

To determine if nondormant seeds can enter secondary dormancy in darkness at temperatures not suitable for germination, nondormant seeds were subjected to various temperature conditions for different durations of time and subsequently tested for germination. Nondormant seeds were obtained by placing freshly matured seeds into 36 nylon bags (containing seeds only, without sand), then burying six bags in each of six metal boxes filled with wet sand as described above and keeping them at a warm stratification temperature of 30/20 °C for 4 months, at which time seeds were nondormant. Subsequently, one box of nondormant seeds was transferred to 5 °C, 15/5 °C, 20/10 °C, 25/15 °C, 30/20 °C, and 35/25 °C for an additional 5 months. After 0, 1, 2, 3, 4, and 5 months, one bag was removed from each temperature/box, and germination was tested in light at 5 °C, 15/5 °C, 20/10 °C, 25/15 °C, 30/20 °C, and 35/25 °C.

### 2.6 Seasonal changes in seed dormancy status

To test the effects of seasonal temperature fluctuations on seed germinability, 2000 seeds were placed into each of 30 nylon bags and buried at a depth of 2 cm in plastic pots (20 cm diameter × 19 cm height) with drainage holes. These pots containing sterilized soil that had been autoclaved at 121 °C and 15 psi for 3 h to kill seeds of other species were buried in the experimental garden at Yangzhou University (32°23′20″N, 119°25′9″E; elevation 10 m), less than 2 km from the seed collection site, beginning on 20 May 2022. The tops of the pots were level with the soil surface. After burial, soil in the pots was watered to field capacity; thereafter, no additional irrigation was provided, leaving the seeds exposed to natural rainfall. Daily maximum and minimum air temperatures were recorded throughout the study period.

From June 2022 to November 2024, one bag was retrieved at the end of each month from a randomly selected pot. Any germinated seedlings were counted and discarded, and the remaining seeds were subjected to germination tests under both light and dark conditions at 5 °C, 15/5 °C, 20/10 °C, 25/15 °C, 30/20 °C, and 35/25 °C, as described above. All manipulations for seeds incubated in the dark, including retrieving pots from the field and counting seeds in the laboratory, were conducted at night under a green “safe light,” which does not promote seed germination (Baskin and Baskin, 2014), to ensure that the seeds were never exposed to white light from burial to the end of the germination tests.

### 2.7 Germination phenology

To monitor germination phenology of *Cardamine* *impatiens* seeds in the field, four replicates of 100 fresh seeds each were sown on the soil surface in plastic pots (20 cm in diameter × 19 cm in height, with drainage holes) on May 20, 2022. The seeds were placed on the surface because previous studies indicate that seeds of some winter annual species cannot germinate in the dark (*e.g*., Baskin and Baskin, 1989a; Soltani et al., 2016). Prior to sowing, the soil in the pots was irrigated to field capacity and then watered weekly throughout the experiment, except during the winter months. The pots were placed in a non-heated greenhouse without artificial heating or cooling and windows kept open year-round, thereby exposing the seeds to natural seasonal temperature fluctuations. Germination was checked weekly, and any germinated seeds were counted and removed. The cumulative percentage of germinated seeds was calculated monthly. Daily maximum and minimum air temperatures were recorded throughout the study period. Over the 30 months of the study, daily maximum and minimum temperatures in the field experimental garden were highly correlated with those in the non-heated greenhouse (both *r* > 0.99, *p* < 0.0001), with the mean maximum temperature in the greenhouse 0.15 °C lower and the mean minimum temperature 0.54 °C higher than in the field.

### 2.8 Data analysis

We tested the effects of various treatments (*i.e.,* dry storage period, stratification temperature, germination temperature) and their interactions on seed germination using General Linear Models (GLMs). As an initial exploratory analysis, we selected three error distributions: binomial with a logit link, Poisson, and Gaussian. We used the DHARMa package (Hartig, 2020) to diagnose the residuals by examining the QQ plot residuals and the residual-vs-predicted plots. We found that although the suitable error distribution differed for each analysis, the results were consistent across these distributions, and all treatments and their interactions were statistically significant. Hence, to ensure model consistency we selected the default Gaussian error distribution for the formal analysis (Tables S1–S5). In the dry storage study, no seeds germinated under dark conditions; therefore, the dark treatment was excluded to prevent distortion of the residual distributions and violation of model assumptions (Table S1). For all analyses, the significance of terms in the GLM was further assessed using analysis of variance (ANOVA), and statistically significant (*p* < 0.05) main effects and interactions were further analyzed using HSD post hoc tests. All data analyses were conducted using R software (v.4.2.2, R Core Team, 2022).

## 3 Results

### 3.1 Effects of light and dry storage period (afterripening) on seed germination

**Fig. 1:**
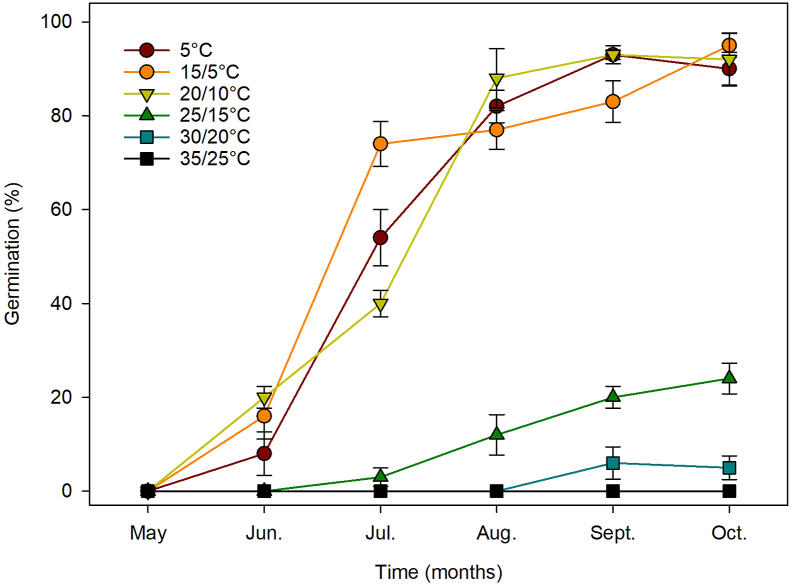
Germination percentages (mean ± s.e.) of *Cardamine impatiens* seeds incubated under six temperature regimes in light (12 h photoperiod) after dry storage at ambient room temperature from May (fresh seeds) to October (after 5 months of dry storage). No seeds germinated in darkness after any duration of dry storage.

After 2 weeks of incubation in both light and dark at 5 °C, 15/5 °C, 20/10 °C, 25/15 °C, 30/20 °C, and 35/25 °C, no freshly matured seeds had germinated, indicating that they were dormant. However, with the extension of the dry storage period, seeds gradually gained the ability to germinate in light at low temperatures (5 °C, 15/5 °C, and 20/10 °C), while germination in light at high temperatures (25/15 °C, 30/20 °C, and 35/25 °C) remained < 25% (Fig. 1 and Table S1), and no seeds germinated in the dark at any temperature.

### 3.2 Temperature requirement for dormancy break

**Fig. 2:**
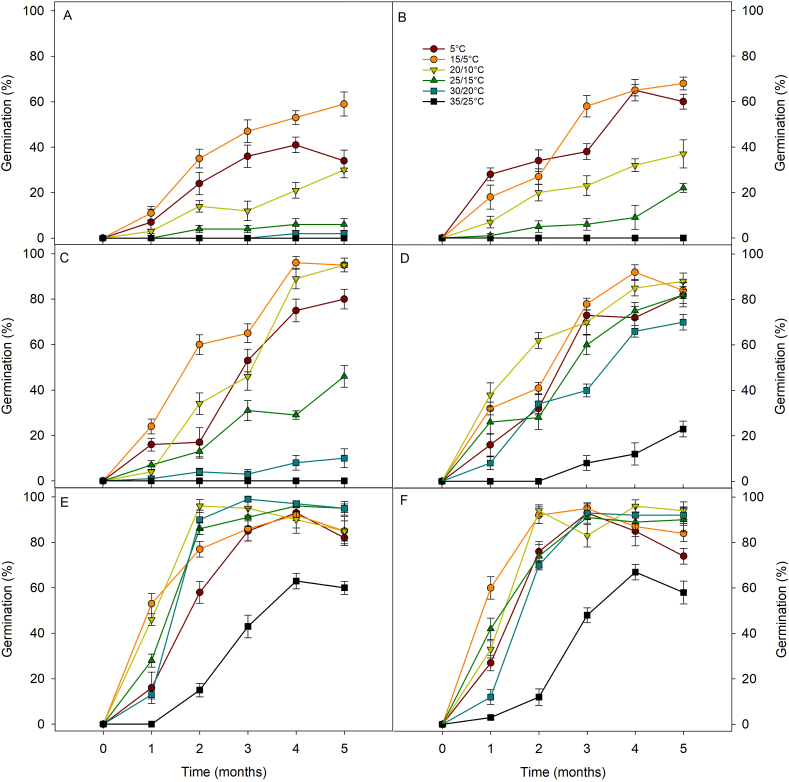
Germination percentages (mean ± s.e.) of *Cardamine impatiens* seeds incubated under different temperature regimes in light (12 h photoperiod) after stratification for various durations of time at (A) 5 °C, (B) 15/5 °C, (C) 20/10 °C, (D) 25/15 °C, (E) 30/20 °C, and (F) 35/25 °C.

With an increase in seed stratification period and/or stratification temperature, germination percentages increased, and the thermal breadth for germination widened (Fig. 2 and Table S2). When seeds were stratified at low temperatures, they gradually gained germinability at low temperatures, but not at high temperatures (e.g., Fig. 2A and B). In contrast, when seeds were stratified at high temperatures, they gained the ability to germinate both at low and high temperatures. For example, after 5 months of stratification at 30/20 °C or 35/25 °C, > 80% of seeds germinated at all tested temperatures except at 35/25 °C, where about 60% seeds germinated (Fig. 2E and F).

### 3.3 Temperature requirement for induction of secondary dormancy

**Fig. 3:**
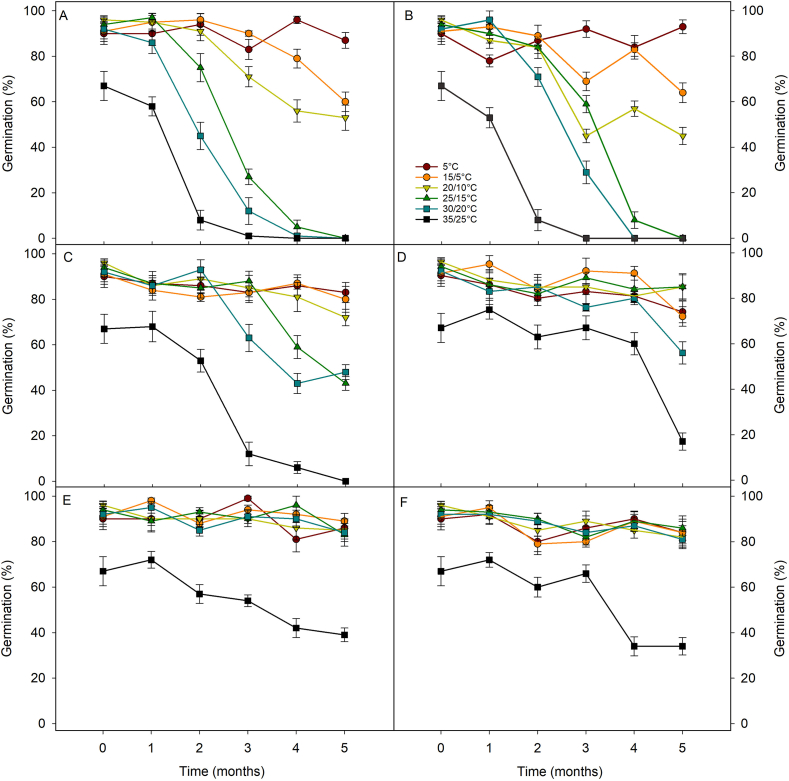
Germination percentages (mean ± s. e.) of *Cardamine impatiens* seeds stratified in dark for 4 months at 30/20 °C, then transferred to (A) 5 °C, (B) 15/5 °C, (C) 20/10 °C, (D) 25/15 °C, (E) 30/20 °C, and (F) 35/25 °C for different durations of time, followed by incubation under various temperature regimes in light (12 h photoperiod).

When seeds of *Cardamine* *impatiens* were stratified for 4 months at 30/20 °C, they germinated to ≥ 90% in light at 5 °C, 15/5 °C, 20/10 °C, 25/15 °C, and 30/20 °C, and to 67% at 35/25 °C (Fig. 3, 0 months). However, when nondormant seeds were transferred to lower temperatures such as 5 °C (Fig. 3A) and 15/5 °C (Fig. 3B), germination at high temperatures declined sharply, while germination at low temperatures remained relatively high (Fig. 3 and Table S3). In contrast, when seeds were transferred to higher temperatures, such as 30/20 °C (Fig. 3E) or 35/25 °C (Fig. 3F), germination decreased only at 35/25 °C but not at other temperatures, and the decrease was less pronounced than that of seeds transferred to lower temperatures.

### 3.4 Seasonal changes in seed dormancy status

**Fig. 4:**
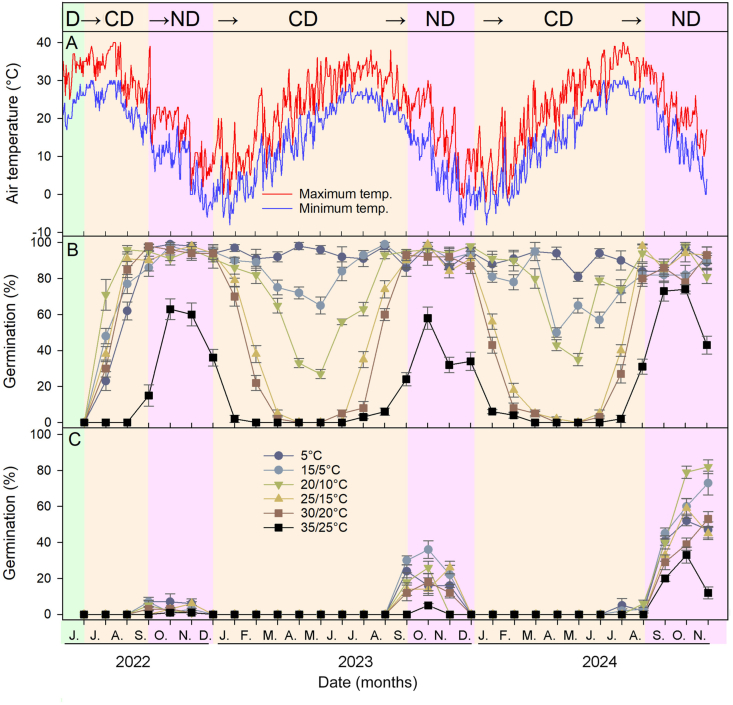
(A) Mean daily maximum and minimum air temperatures at the sites where seeds were buried. (B, C) Germination percentages (mean ± s. e.) of *Cardamine impatiens* seeds incubated under different temperature regimes in light (12 h photoperiod) (B) and darkness (C) following 0–30 months of burial. Fresh *C. impatiens* seeds were buried in nylon bags at a soil depth of 2-cm in the field in May 2022 and exhumed at monthly intervals. Germination tests were conducted at the end of each month. D: dormancy, CD: conditional dormancy, ND: non-dormancy.

Seeds of *C. impatiens* buried in the field exhibited clear seasonal changes in their germinability under light across the temperature regimes tested (Fig. 4 and Table S4). Seeds were dormant at burial, and their dormancy was gradually broken in July–August 2022, when the monthly maximum and minimum air temperatures were > 34 °C and 26 °C, respectively (Fig. 4A), with germination at high temperatures being generally slightly lower than those at low temperatures (Fig. 4B). By September–October 2022, as temperatures declined, seeds achieved maximum germination across all tested temperatures. However, from December 2022 to March 2023, the buried seeds gradually lost germinability at high (but not at low) temperatures (Fig. 4B), and by April–May, no seeds germinated at 25/15 °C, 30/20 °C, or 35/25 °C. Thereafter, seeds maintained their high germinability at 5 °C, 15/5 °C, and 20/10 °C throughout the year until the end of the experiments, while germinability at 25/15 °C, 30/20 °C, and 35/25 °C exhibited a similar cyclical pattern to 2022 (Fig. 4B).

Seeds germinated in darkness also showed seasonal changes. However, compared to seeds germinated under light, germination in darkness was significantly lower (Fig. 4C). Moreover, seeds exhibited germinability only between September and November. In addition, germination percentages in darkness increased significantly with each successive year of burial. In 2022, ≤ 7% of the seeds germinated, whereas 36% and 80% of the seeds germinated in darkness in 2023 and 2024, respectively, with the highest percentages observed at 5 °C, 15/5 °C, and 20/10 °C (Fig. 4C). Note that during the 30 months of burial, seed viability remained high, with at least 85–90% of the nongerminated seeds still viable, and the loss in viability was partly attributed to a small portion (< 10%) that germinated in the field, leaving empty seed coats or dead radicles.

### 3.5 Germination phenology

**Fig. 5:**
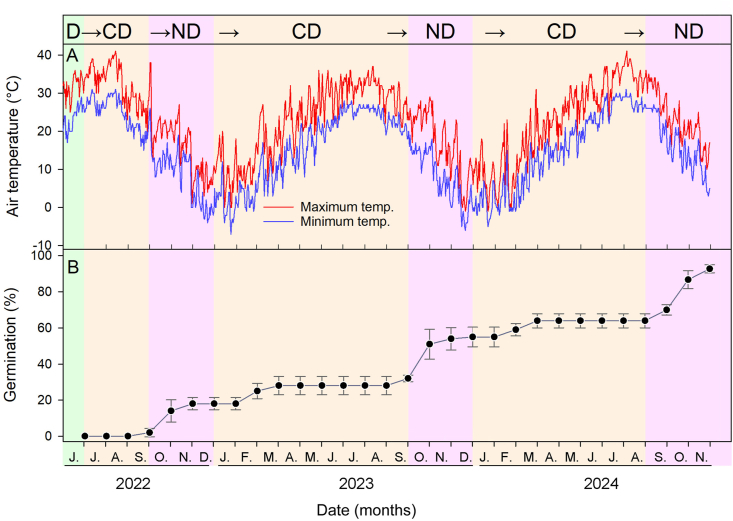
(A) Mean daily maximum and minimum air temperatures in a non-heated greenhouse where seeds were buried. (B) Germination phenology (percentages, mean ± s.e.) of *Cardamine impatiens* seeds sown on the surface of the soil in the non-heated greenhouse. D: dormancy, CD: conditional dormancy, ND: non-dormancy.

Germination of seeds exposed on the soil surface occurred both in autumn and spring during the 3 years of the study (Fig. 5 and Table S5). In 2022, germination primarily occurred in October (14%), with mean monthly maximum and minimum temperatures of 21.6 °C and 13.1 °C, respectively, while smaller percentages were observed in September (2%) and November (4%), resulting in an overall autumn germination of 20%. In 2023, the accumulated germination was 36%, with 10% in February and March (spring), when the mean monthly maximum and minimum temperatures were > 9.43 °C and 2.82 °C, respectively, and 26% in autumn, of which 19% occurred in October, when temperatures were 24.1 °C and 14.8 °C. In 2024, 9% of seeds germinated in February and March, while 40% germinated in October and November (Fig. 5).

## 4 Discussion

Freshly matured seeds of *Cardamine* *impatiens* were dormant (Fig. 1), and dormancy-break of buried seeds occurred during summer, with seeds first gaining the ability to germinate at low and then gradually at relatively high temperatures (Fig. 2, Fig. 4). However, the high temperatures of summer inhibited germination (*i.e.*, seeds in CD). By autumn, when daily temperatures had declined, the seeds were ND and could germinate to high percentages over a range of temperatures in light, with little or no germination in darkness (Fig. 4B). Seeds that did not germinate in autumn entered CD when exposed to the low temperatures of winter and lost germinability at high but not at low temperatures, thereby allowing a narrow germination window in early spring (Fig. 3, Fig. 4, Fig. 5). During the following summer, CD was gradually released, *i.e.*, seeds of a given cohort in the soil experience seasonal cycles of CD and ND until the cohort of seeds is depleted. These findings are generally consistent with our predictions 1–4; hence, our hypothesis is supported.

### 4.1 Temperature requirement for dormancy break

Baskin and Baskin (2021; also see Soltani et al., 2017) divided seeds with nondeep physiological dormancy into six types (types 1–6), among which seeds with Type 1 initially gain the ability to germinate at low temperatures. Then, with an increase in dormancy-breaking conditions the maximum temperatures at which seeds can germinate increases (Soltani et al., 2017, 2019; Baskin and Baskin, 2021). Both dry storage (Fig. 1) and stratification at various temperatures (Fig. 2) promoted dormancy-break of *Cardamine* *impatiens* seeds, during which seeds gained germinability first at low temperatures and then expanded to include higher temperatures. Thus, seeds of this species have Type 1 nondeep physiological dormancy. This is consistent with the kind of dormancy found in seeds of other Brassicaceae species (*e.g*., Karimmojeni et al., 2014; Soltani et al., 2016; Lu et al., 2017; Maleki et al., 2021, but see Guo et al., 2025) and with that in seeds of winter annuals in many other families (*e.g*., Copete et al., 2009; Baskin and Baskin, 2014).

Five months of dry storage (from late May to October) facilitated germination of *Cardamine* *impatiens* seeds only at 5 °C, 15/5 °C, and 20/10 °C, the temperatures typical of late October and November. Similarly, low-temperature stratification (*e.g*., Fig. 2A and B), corresponding to winter, resulted in the highest germination at 5 °C and 15/5 °C, the temperatures typical of early spring. This partial dormancy-break, at low temperatures, has been found in seeds of other species of winter annuals, including two species of Brassicaceae: *Arabidopsis thaliana* and *Cardamine hirsuta* (Baskin and Baskin, 2014). Although some dormancy-break can occur at a low temperature (*e.g*., 5 °C), this is unlikely to occur in the field because newly-dispersed seeds of *C. impatiens* first are exposed to high temperatures of summer, which break dormancy.

High-temperature (*e.g*., 30/20 °C or 35/25 °C) stratification is more effective in dormancy-break than low-temperature stratification or dry storage (Fig. 2), which is also consistent with the environment seeds are likely to experience after dispersal. However, in the field, seeds that experienced temperatures from June to August still did not expand their upper thermal threshold for germination to include these high summer temperatures (Fig. 4B). In other words, germination in the field was inhibited during summer because the sustained high temperatures (35/25 °C) in July and August exceeded their upper thermal threshold, thus preventing germination. Such thermoinhibition has been interpreted as an adaptive response that reduces the risk of seedling mortality due to summer heat (Footitt et al., 2021), particularly since Brassicaceae species are generally vulnerable to heat stress (Hasanuzzaman, 2020; Cantila et al., 2024). Nevertheless, seeds acquire the ability to germinate at 35/25 °C in September and October. This response appears maladaptive, however, since those high temperatures have already passed in the field by that time (Fig. 2, Fig. 4B).

### 4.2 Temperature requirement for induction of secondary dormancy

In the field, a small amount of germination was observed in September followed by a major increase in October (Fig. 5). At this time, seed dormancy had been broken, and seeds germinated under all tested temperature regimes in light (Fig. 4B). Thereafter, as temperatures declined with the onset of winter seeds failed to germinate because temperatures were below their lower thermal threshold, and buried seeds were in the dark. Additionally, prolonged cold during winter caused seeds that could previously germinate across a broad temperature range to lose germinability at high temperatures while retaining it at lower temperatures (Fig. 4B). Consequently, seeds may germinate in the field only during early spring if light is not limiting (Fig. 5).

### 4.3 Light requirement for germination

Darkness inhibited germination of *Cardamine* *impatiens* seeds, and such light-dependent germination is common among winter annual species, ensuring that seeds emerge near the soil surface, thereby reducing the risk of energy depletion from deep burial (Chauhan et al., 2006). Furthermore, such a light requirement for seed germination of *C. impatiens*, coupled with high seed viability after 30 months of burial, indicates the presence of a persistent soil seed bank (Walck et al., 2005). However, despite this initially strict light requirement for germination seeds progressively gained the ability to germinate in darkness after prolonged burial (Fig. 4C). This increased germination in darkness also has been observed in the Brassicaceae obligate winter annual species *Lesquerella lescurii* (Baskin et al., 1992). The implication of increased ability to germinate in darkness is that (if not re-supplied) the soil seed bank may be depleted over time by *in situ* germination.

### 4.4 Dormancy cycling as a functional trait and its ecological significance

After innate dormancy was broken during summer, buried seeds of *Cardamine* *impatiens* exhibited cycling between ND (autumn to early winter) when they germinated across a broad range of temperatures in light and CD (winter to summer) when they germinated only at low (early spring) temperatures in light. A similar CD↔ND cycle has also been observed in Brassicaceae facultative winter annuals like *Capsella bursa-pastoris* (Baskin and Baskin, 1989a). Compared with seeds of the obligate winter annuals *Arabidopsis thaliana* (Baskin and Baskin, 1983) which exhibit a D↔CD↔ND cycle with a single (autumn) annual germination window, the seeds of *C. impatiens* have two potential germination windows: autumn and early spring. Furthermore, since seeds of *C. impatiens* cannot germinate in late spring, which ensures that seedlings/plants are not exposed to the hot summer conditions that are often detrimental to Brassicaceae winter annual species (Franks, 2011; Hasanuzzaman, 2020; Cantila et al., 2024). As such, dormancy cycling in *C. impatiens* and probably other facultative winter annuals could be considered an adaptive functional trait (Violle et al., 2007), *i.e*., it mitigates the risk of catastrophic recruitment failure and allows plants to establish under conditions that maximize their fitness, thereby potentially enhancing long-term population persistence.

As climate change intensifies seasonal unpredictability, functional traits that enable plants to cope with such fluctuating climatic conditions become increasingly important (Reich et al., 2003). We suggest that dormancy cycling is one such trait that enables seeds to track unpredictable shifts, and reduces the risk of premature emergence and mortality during unfavorable periods. Since seeds of *Cardamine* *impatiens* require high summer temperatures to break dormancy, climate warming is unlikely to negatively affect dormancy break in this species. However, a warming climate may delay seedling emergence while increasing seed yield in the autumn cohort, as evidenced by studies on obligate winter annual species (Footitt et al., 2021; Batlla et al., 2022), and advance seedling emergence in the spring cohort, as observed in many summer annual species (*e.g*., Milbau et al., 2009). Hence, under climate change scenarios, facultative winter annual species like *C. impatiens* may exhibit greater adaptability than obligate winter annuals and summer annuals, at least from the perspective of seed ecology.

Nevertheless, dormancy cycling complicates management of weedy or invasive species, such as *Cardamine* *impatiens*. Because seeds can establish in both autumn and spring, single-season interventions are insufficient and may inadvertently promote germination by disturbing the soil. Therefore, effective management requires a multi-pronged strategy that targets both autumn and spring germination windows and reduces the soil seed bank. Effective approaches may involve consistently removing plants before seed set and preventing further replenishment of the seed bank, thereby reducing the population over time.

### 4.5 Possible limitations and future perspectives

Germination timing is a niche-constructing trait that modifies the conditions under which plants develop (Donohue et al., 2005). However, without fitness-related data (*e.g*., seedling survival or reproductive success) it remains unclear whether the absence of summer germination reflects an adaptive response to summer stressors or a constraint imposed by physiological limitations, and to what extent dormancy cycling optimizes reproductive success. Hence, more studies linking germination phenology (*e.g*., early and late autumn, as well as early and late spring) to fitness outcomes in the field such as the one by Lu et al. (2014) are required to clarify the ecological and evolutionary significance of these responses.

In addition, our study focused on a single population of this widely distributed species and did not distinguish between seeds derived from autumn and spring cohorts. Future research examining multiple populations from different climatic regions and differentiating between autumn- and spring-derived seeds would provide a more comprehensive understanding of how climate conditions might shape the germination behaviors of *C. impatiens*. Finally, future studies exploring how physiological costs influence seed persistence and population dynamics, especially under shifting climatic regimes, are needed to evaluate the trade-offs between seed persistence in the seed bank and plant fitness.

## 5 Conclusions

**Fig. 6:**
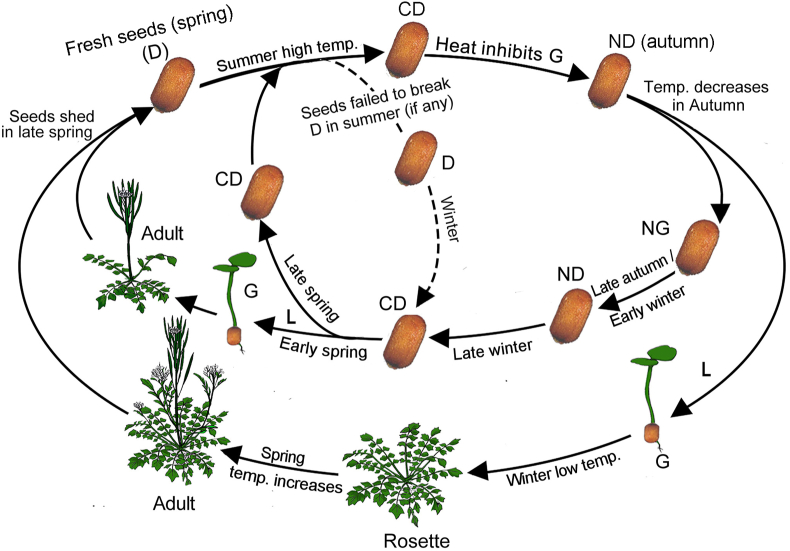
A conceptual model illustrating the dynamics of dormancy and germination in *Cardamine impatiens* seeds within the soil seed bank. D: innate (or true) dormancy; CD: conditional dormancy; ND: non-dormancy; NG: non-germination; G: germination; L: seeds that germinated were exposed to light, *e.g*., they were brought to the soil surface by disturbance.

The results of our study can be summarized in a conceptual model (Fig. 6). Fresh seeds of *Cardamine* *impatiens* are dormant at maturity and exhibit an annual conditional dormancy/non-dormancy cycle when buried in soil and exposed to natural seasonal temperature fluctuations. The high temperatures in July and August break seed dormancy but suppress germination, preventing seedlings from emerging in the field during this period. By September and October, temperatures decrease sufficiently to allow germination, and if soil moisture and light are not limiting germination occurs. Seeds that do not germinate in autumn enter conditional dormancy during winter, losing germinability (but retaining viability) at high but not low temperatures. Consequently, seeds of *C. impatiens* germinate in both autumn and spring if exposed to light, but they remain nongerminated while buried. In other words, seeds that fail to germinate in the first autumn following burial (*e.g*., due to insufficient light) may germinate the following spring or in a subsequent autumn or spring if exposed to light. Overall, seeds of *C. impatiens* exhibit adaptive dormancy strategies that integrate environmental signals, such as seasonal temperature fluctuations and light availability, to fine-tune germination timing. These mechanisms ensure that germination occurs during cooler conditions in autumn and early spring, optimizing seedling emergence under favorable conditions while avoiding lethal summer heat. In sum, dormancy cycling likely is an adaptive functional trait that controls timing of germination in the habitat.

## CRediT authorship contribution statement

**Jingshi Yang:** Writing – original draft, Visualization, Investigation, Formal analysis. **Yan Luo:** Writing – original draft, Visualization, Validation, Investigation. **Jerry M. Baskin:** Writing – review & editing, Writing – original draft. **Carol C. Baskin:** Writing – review & editing, Writing – original draft. **Andreas Prinzing:** Writing – review & editing. **Luping Liu:** Writing – original draft, Methodology, Investigation. **Chaohan Xu:** Supervision, Investigation. **Keliang Zhang:** Writing – original draft, Supervision, Methodology, Funding acquisition, Formal analysis, Conceptualization.

## Data availability

The data used in the present study can be accessed at: https://doi.org/10.6084/m9.figshare.28148114.v1

## Declaration of competing interest

The author Carol C. Baskin is an Editorial Board Member for Plant Diversity and was not involved in the editorial review or the decision to publish this article. The other authors declare that they have no known competing financial interests or personal relationships that could have appeared to influence the work reported in this paper.
