# Supplementary material for: Seed dormancy cycling: A driver of germination timing in a facultative winter annual
Source: Plant Divers. 2025 May 27;48(3):618–28. doi: 10.1016/j.pld.2025.05.007 (PMC13250292; doi:10.1016/j.pld.2025.05.007)
Supplement: Multimedia component 1 [file mmc1.docx]

**Supplementary materials for:**

**Seed dormancy cycling: A driver of germination timing in a facultative winter annual**


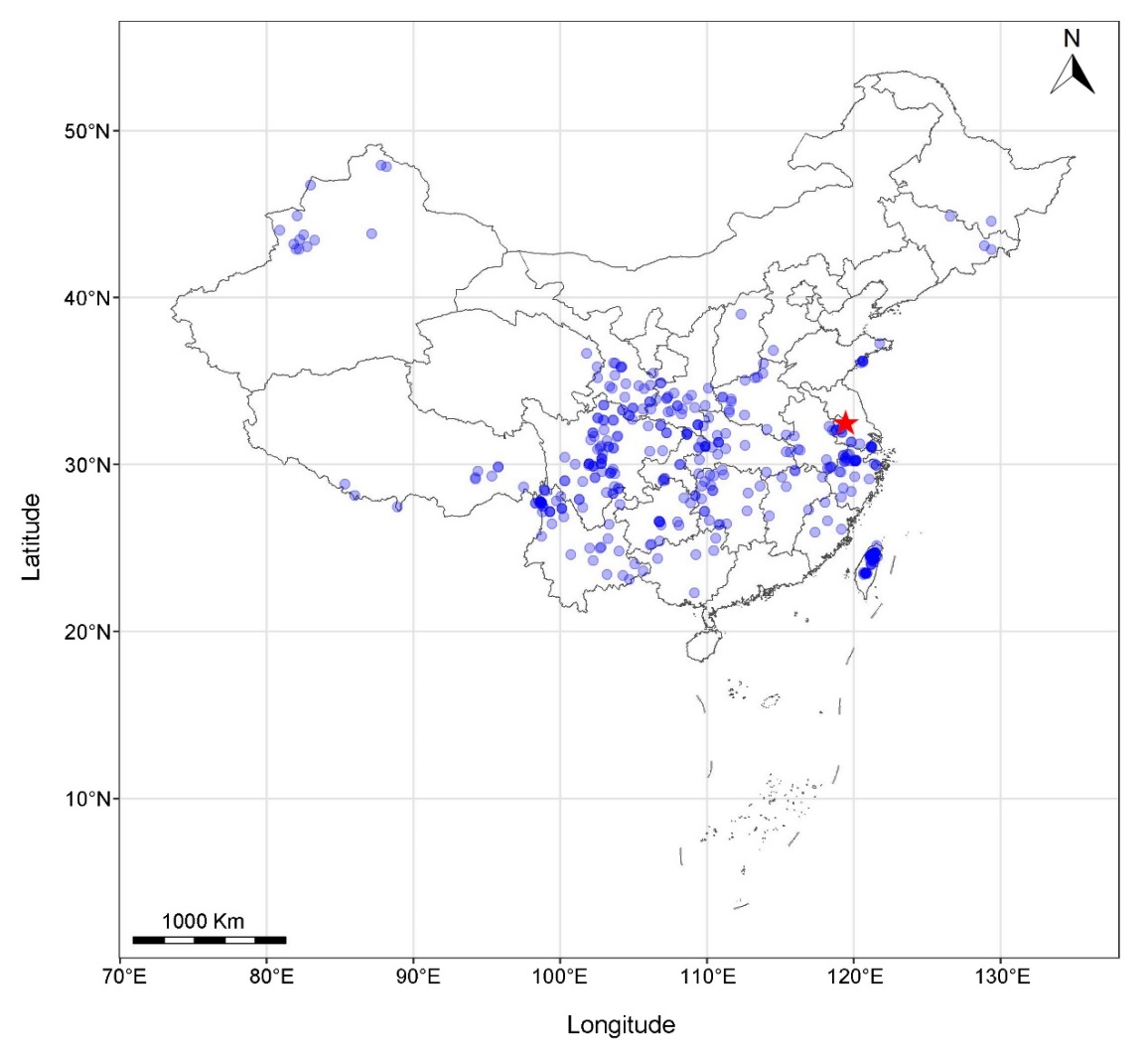


**Fig. S1.** Geographic distribution of *Cardamine impatiens* L. occurrence records (blue points) in China from GBIF (https://www.gbif.org/; accessed on 3 May 2025) and our study site (asterisk). Outlines of provinces and other administrative areas are shown. Note that species occurrence data indicate locations where a plant/specimen has been observed/collected; in other words, the lack of records in certain regions does not necessarily indicate the species cannot occur (is distributed) there.

**Table S1.** Analysis of variance (ANOVA) of the effects of dry storage duration (months) and germination temperature on the germination of *Cardamine impatiens* seeds.

| Source of variation | Sum of Squares | df | Mean Square | *F* | *p* |
| --- | --- | --- | --- | --- | --- |
| Month | 5.7543 | 5 | 1.1509 | 326.0600 | <0.0001 |
| Germination Temperature (GT) | 9.8561 | 5 | 1.9712 | 558.4800 | <0.0001 |
| Month × GT | 4.3793 | 25 | 0.1752 | 49.6300 | <0.0001 |
| Residuals | 0.3812 | 108 | 0.0035 |  |  |

**Table S2.** ANOVA of the effects of stratification temperature, stratification duration, germination temperature, and their full factorial interactions on germination of *Cardamine impatiens* seeds.

| Source of variation | Sum of Squares | df | Mean Square | *F* | *p* |
| --- | --- | --- | --- | --- | --- |
| Stratification Temperature (ST) | 28.9490 | 5 | 5.7900 | 341.2186 | <0.0001 |
| Stratification Duration (SD) | 36.3840 | 1 | 36.3840 | 2144.2615 | <0.0001 |
| Germination Temperature (GT) | 16.0220 | 5 | 3.2040 | 188.8467 | <0.0001 |
| ST × SD | 6.3500 | 5 | 1.2700 | 74.8495 | <0.0001 |
| ST × GT | 5.3480 | 25 | 0.2140 | 12.6064 | <0.0001 |
| SD × GT | 3.1210 | 5 | 0.6240 | 36.7902 | <0.0001 |
| ST × SD × GT | 3.4110 | 25 | 0.1360 | 8.0405 | <0.0001 |
| Residuals | 13.4390 | 792 | 0.0170 |  |  |

**Table S3.** ANOVA of the effects of stratification temperature, stratification duration, germination temperature, and their full factorial interactions on the germination of nondormant seeds of *Cardamine impatiens*. Nondormant seeds were obtained by stratifying fresh seeds for 4 months at 30/20°C.

| Source of variation | Sum of Squares | df | Mean Square | *F* | *p* |
| --- | --- | --- | --- | --- | --- |
| Stratification Temperature (ST) | 8.2558 | 5 | 1.6512 | 254.3156 | <0.0001 |
| Stratification Duration (SD) | 11.6638 | 5 | 2.3328 | 359.2967 | <0.0001 |
| Germination Temperature (GT) | 21.4745 | 5 | 4.2949 | 661.5071 | <0.0001 |
| ST × SD | 5.3477 | 25 | 0.2139 | 32.9464 | <0.0001 |
| ST × GT | 5.3572 | 25 | 0.2143 | 33.0049 | <0.0001 |
| SD × GT | 4.7075 | 25 | 0.1883 | 29.0020 | <0.0001 |
| ST × SD × GT | 6.4078 | 125 | 0.0513 | 7.8955 | <0.0001 |
| Residuals | 4.2072 | 648 | 0.0065 |  |  |

**Table S4.** ANOVA of the effects of burial duration (months), germination light/dark conditions, and germination temperature on germination of *Cardamine impatiens* seeds.

| Source of variation | Sum of Squares | df | Mean Square | *F* | *p* |
| --- | --- | --- | --- | --- | --- |
| Light | 103.3190 | 1 | 103.3190 | 31987.4270 | <0.0001 |
| Month | 38.8930 | 29 | 1.3410 | 415.2170 | <0.0001 |
| Germination Temperature (GT) | 22.7080 | 5 | 4.5420 | 1406.0410 | <0.0001 |
| Light × Month | 16.1300 | 29 | 0.5560 | 172.1990 | <0.0001 |
| Light × GT | 15.7270 | 5 | 3.1450 | 973.8270 | <0.0001 |
| Month × GT | 11.3860 | 145 | 0.0790 | 24.3120 | <0.0001 |
| Light × Month × GT | 14.0340 | 145 | 0.0970 | 29.9660 | <0.0001 |
| Residuals | 3.4880 | 1080 | 0.0030 |  |  |

**Table S5.** ANOVA of the effects of burial duration (months) on the germination of *Cardamine impatiens* seeds.

| Source of variation | Sum of Squares | df | Mean Square | *F* | *p* |
| --- | --- | --- | --- | --- | --- |
| Month | 8.0942 | 29 | 0.2791 | 152.1000 | <0.0001 |
| Residuals | 0.1651 | 90 | 0.0018 |  |  |
